# Influencing factors of biliary fistula after radical resection of hilar cholangiocarcinoma: a prospect cohort

**DOI:** 10.1186/s40001-022-00851-4

**Published:** 2022-10-28

**Authors:** Dengyong Zhang, Feiyu Qi, Wanliang Sun, Guanru Zhao, Dongdong Wang, Shuo Zhou, Zhong Liu, Zheng Lu

**Affiliations:** Department of General Surgery, The First Affiliated Hospital of Bengbu Medical College, No. 287, Changhuai Road, Zhihuai Street, Longzihu District, Bengbu, 233000 Anhui Province China

**Keywords:** Biliary fistula, Hilar cholangiocarcinoma, Surgery, Influencing factors, Treatment

## Abstract

**Background:**

Biliary fistula is a common but serious complication after radical resection of hilar cholangiocarcinoma. We aimed to evaluate the influencing factors of biliary fistula after radical resection, to provide insights to the clinical treatment of hilar cholangiocarcinoma.

**Methods:**

Patients undergoing radical resection of hilar cholangiocarcinoma from January 1, 2015 to March 31, 2022 were selected. Patients’ personnel characteristics and laboratory test results of patients with and without biliary fistula were collected and compared. Logistic regression analyses were conducted to evaluate the associated risk factors of biliary fistula.

**Results:**

160 patients undergoing radical resection of hilar cholangiocarcinoma were included, the incidence of postoperative biliary fistulas was 20.63%. There were significant differences in the age, preoperative cholangitis and number of biliary anastomosis between biliary fistula and no biliary fistula patients (all *p* < 0.05). There were significant differences in the gamma glutamyl transpeptidase (GGT) on the first day after surgery, Klebsiella pneumoniae between biliary fistula and no biliary fistula patients (all *p* < 0.05). Logistic regression analysis indicated that age ≥ 65 years (OR  2.035, 95%CI 1.131–3.007), preoperative cholangitis (OR 1.584, 95% CI 1.081–2.361), number of biliary anastomosis ≥ 2(OR 2.866, 95%CI 1.942–3.624), GGT on the first day after surgery ≥ 120 U/L (OR 1.823, 95%CI: 1.274–2.906), preoperative bile culture for Klebsiella pneumoniae (OR 3.181, 95%CI: 2.426–3.992) were the risk factors of postoperative biliary fistulas (all *p* < 0.05).

**Conclusions:**

There are many independent risk factors for postoperative biliary fistula in patients undergoing radical resection of hilar cholangiocarcinoma. Clinical medical workers should take early interventions and treatment measures for these high-risk patients to reduce the occurrence of postoperative biliary fistula.

## Introduction

Hilar cholangiocarcinoma is the most common malignant tumor of the biliary tract, which has the characteristics of special location, insidious onset, and complex structure [1, 2]. Diagnosis and treatment of hilar cholangiocarcinoma have always been one of the difficult problems in surgery. In recent years, the incidence of hilar cholangiocarcinoma has been on the rise [3, 4]. With the advancement of imaging and hepatobiliary surgery technology, the surgical resection rate and prognosis of the disease have been significantly improved. The concept of surgical treatment for the disease tends to expand the scope of resection and to achieve the effect of radical tumor resection [5]. Especially for the common Bismuth type IIIa, IIIb and IV, it is often necessary to combine left and right liver and caudate lobe resection to achieve negative resection margins and achieve the effect of radical tumor resection [6]. The invasion of surrounding tissues and organs requires combined vascular resection and reconstruction or pancreaticoduodenectomy [7]. Therefore, the safety and effect of resection of hilar cholangiocarcinoma have been the focus of surgeons.

In recent years, despite the continuous improvement of surgical skills and perioperative management, the incidence of biliary fistula radical resection of hilar cholangiocarcinoma can up to 39.16%, which is much higher than that of simple hepatectomy (4.04–16.85%) [8–10]. The occurrence of postoperative biliary fistula will lead to prolonged hospital stay, delayed adjuvant therapy, increased economic burden and perioperative mortality [11–13]. Some studies [14, 15] have pointed out that postoperative biliary fistula can also affect the long-term survival of patients. According to the definition and grading of postoperative biliary fistula by the International Study Group of Liver Surgery (ISGLS) [16], grade A biliary fistula has a short course (< 7 days) and hardly affects the postoperative management of patients, while biliary fistulas of grades B and C are closely related to the clinical situation, which usually means a protracted disease course or a change in treatment decisions. Therefore, early identification of grade B and C biliary fistulas and effective intervention are of great clinical significance to the progress of postoperative patients with hilar cholangiocarcinoma. Currently, the influencing factors of biliary fistula after radical resection of hilar cholangiocarcinoma remain unclear. Therefore, we prospectively analyzed the characteristics and laboratory test results of patients undergoing radical resection of hilar cholangiocarcinoma in our hospital, to analyze the risk factors of clinically relevant biliary fistula after radical resection of hilar cholangiocarcinoma, to provide reliable evidence to the clinical management and treatment of hilar cholangiocarcinoma.

## Methods

### Ethics

In this study, all methods were performed in accordance with the relevant guidelines and regulations. This study was a prospective cohort design. This present study had been verified and approved by the ethical committee of The First Affiliated Hospital of Bengbu Medical College with approval number: 2021230. And written informed consents had been obtained from all the included patients.

### Patients

We selected patients who underwent radical resection of hilar cholangiocarcinoma in our hospital from January 1, 2015 to March 31, 2022 as the research population. The inclusion criteria of patients were as follows: (1) the pathological results indicated the diagnosis of hilar cholangiocarcinoma; (2) patients received radical surgery including partial hepatectomy, extrahepatic bile duct resection, regional lymph node dissection and Roux-en-Y biliary anastomosis performed by the same group of surgeons; (3) the patients had complete clinical and pathological data; (4) patients agreed to participate in this study. The exclusion criteria of patients were as follows: (1) patients with incomplete data; (2) patients did not agree to participate in this study.

### Patient management

After admission, the clinical diagnosis and treatment plan were determined by multidisciplinary discussion of hepatobiliary and pancreatic diseases. The patient's nutritional status was reassessed weekly and nutritional support regimens adjusted. For patients with preoperative infection, the operation should be performed after the infection has been effectively controlled (normal body temperature > 72 h, clinical symptoms are relieved, and the results of routine laboratory tests were in the normal range). Prophylactic antibiotic ceftriaxone sodium 2 g was routinely given 30 min before surgery.

All the included patients received the same surgical procedure, and the surgery was performed by the same group of surgeons. Preoperative laparoscopic exploration was performed to assess resectability in patients with peritoneal carcinomatosis. All patients underwent partial hepatectomy, extrahepatic bile duct resection, regional lymph node dissection, No. 9 and No. 16 lymph node biopsies, and Roux-en-Y cholangiojejunostomy. Anatomical hepatectomy was used during the operation. For the blood vessels of the preserved side liver, fine dissection and suspension protection were routinely performed during the operation. Before the end of the operation, the blood flow was routinely checked by intraoperative Doppler ultrasonography. During liver incision, the Pringle method was used to block the blood flow into the liver. The ultrasonic suction device combined with the fine clamp method was used to cut off the liver parenchyma. During the intraoperative assessment of the edge of the hepatic duct resection, additional duct resections were performed to achieve R0 resection. The pipe structures with a diameter of < 0.5 cm were clamped with titanium clips, and the pipe structures with a diameter of > 0.5 cm were clamped with titanium clamps. The oxidized cellulose was used to close the cut plane of the liver parenchyma. The bile duct and the jejunum bridge were sutured end-to-side with 5-0/6-0 PDS sutures for continuous cholangioenteric anastomosis. The liver section was carefully examined without active bleeding or biliary fistula.

Biochemical, bacterial smear and culture examinations were performed on the peritoneal drainage fluid on the 1st, 3rd, and 7th days after the operation, and blood biochemical examinations were performed at the same time. Follow-up monitoring was performed at least once a week until the peritoneal drainage tube was removed. Biochemical tests were performed immediately when bile-like fluid was observed to drain from the drain. For patients with postoperative biliary fistula, the formulation and change of treatment measures were ultimately decided by the multidisciplinary discussion of our medical teams according to the conditions of patient.

### Observation indicator

The diagnostic criteria for biliary fistula were that after 3 days after operation, the total bilirubin (TBil) of peritoneal drainage fluid was > 3 times the serum level of the same period, or intervention or secondary surgery was required due to biliary peritonitis [16, 17]. According to the ISGLS definition [15], bile leakage was diagnosed and graded into A, B and C levels based on severity. The diagnostic criteria for cholangitis were systemic inflammation, cholestasis, and imaging evidence of at least one each. Systemic inflammatory manifestations including: (1) fever (body temperature > 38 °C) and/or chills; (2) laboratory support, including white blood cell (WBC) count < 4 × 10^9^/L or > 10 × 10^9^/L, C-reactive protein (CRP) > 10 mg/L [18].

We collected patients’ personnel characteristics including: gender, age, body mass index (BMI), hypertension, diabetes, Bismuth type, time of intraoperative liver ischemia based on the Pringle method, preoperative cholangitis, American Society of Anesthesiologists (ASA) score, number of biliary anastomosis, estimated blood loss during surgery, blood infusion during surgery, duration of surgery. Besides, we collected the laboratory test results before surgery and on the first day after surgery, including platelet count (PLT), alanine aminotransferase (ALT), aspartate aminotransferase (AST), alkaline phosphatase (AKP), gamma glutamyl transpeptidase (GGT), TBil, cholinesterase (ChE), international normalized ratio(INR). Additionally, we collected the preoperative bile culture results.

### Statistical analysis

SPSS 22.0 statistical software was used for statistical analysis of data in this study. Quantitative data with normal distribution were described as mean ± standard deviation, and t-test was used for comparison. Quantitative data that were not normally distributed were expressed as medians (interquartile range) and compared using the rank sum test. The enumeration data were expressed as absolute numbers, and the comparison was performed by *χ*2 test, likelihood ratio test or Fisher's exact test according to the applicable conditions. Univariate and multivariate analyses of clinically relevant risk factors for biliary fistula were performed using logistic regression models. Combined with clinical application, variables with univariate analysis *p* < 0.05 were included in multivariate analysis. Receiver operating characteristic (ROC) curves were drawn and the diagnostic value of the model was analyzed by calculating the area under the curve (AUC). In this study, *p* < 0.05 was considered as a statistically significant difference between groups.

## Results

### The characteristics of included patients

A total of 160 patients undergoing radical resection of hilar cholangiocarcinoma were included, of whom 33 patients had postoperative grade B and C biliary fistulas, the incidence of postoperative biliary fistulas in patients undergoing radical resection of hilar cholangiocarcinoma was 20.63%. As shown in Table 1, there were significant differences in the age, preoperative cholangitis and number of biliary anastomosis between biliary fistula and no biliary fistula patients (all *p* < 0.05). There were no significant differences in the gender, BMI, hypertension, diabetes, Bismuth type, time of intraoperative liver ischemia, ASA score, estimated blood loss during surgery, blood infusion during surgery, duration of surgery between biliary fistula and no biliary fistula patients (all *p* > 0.05).

**Table 1:** The characteristics of included patients (*n* = 160)

Variables	Biliary fistula group (*n* = 33)	No biliary fistula group (*n* = 127)	t/χ^2^	p
Male/female	20/13	84/43	1.041	0.069
Age (y)	69.86 ± 10.22	57.02 ± 9.34	3.088	0.011
BMI (kg/m^2^)	21.01 ± 2.13	21.85 ± 2.02	2.904	0.061
Hypertension	10(30.30%)	34(26.77%)	2.131	0.077
Diabetes	9(27.27%)	36(28.35%)	1.918	0.083
Bismuth type			1.557	0.067
I	0(0%)	3(2.36%)		
II	4(12.12%)	17(13.39%)		
IIIa	7(21.22%)	38(29.92%)		
IIIb	11(33.33%)	40(31.49%)		
IV	11(33.33%)	29(22.83%)		
Preoperative cholangitis	12(36.36%)	26(20.47%)	1.466	0.013
Time of intraoperative liver ischemia(min)	46.26 ± 19.10	42.09 ± 16.64	11.26	0.061
ASA score ≥ 3	24(72.73%)	78(61.42%)	2.051	0.071
Number of biliary anastomosis	2.88 ± 0.76	1.12 ± 0.22	1.849	0.035
Estimated blood loss during surgery	625.49 ± 285.05	588.14 ± 255.38	42.021	0.115
Blood infusion during surgery	25(75.76%)	92(72.44%)	1.261	0.072
Duration of surgery(days)	618.04 ± 124.92	601.29 ± 131.54	35.073	0.149

### Laboratory test results

As shown in Table 2, there was significant difference in the GGT on the first day after surgery between biliary fistula and no biliary fistula patients (*p* = 0.025). There were no significant differences in the PLT, ALT, AST, AKP, TBil, ChE and INR before surgery and on the first day after surgery between biliary fistula and no biliary fistula patients (all *p* > 0.05).

**Table 2:** Comparison of laboratory test results before surgery and on the first day after surgery

Variables	Biliary fistula group (*n* = 33)	No biliary fistula group (*n* = 127)	*t*	*p*
Before surgery				
PLT(× 10^9^/L)	245.01 ± 78.84	238.14 ± 73.02	17.192	0.145
ALT(U/L)	62.82 ± 44.15	65.33 ± 48.24	8.024	0.095
AST(U/L)	39.96 ± 20.02	40.26 ± 19.41	5.038	0.244
AKP(U/L)	208.47 ± 85.13	195.21 ± 90.46	26.107	0.277
GGT(U/L)	252.08 ± 99.18	218.85 ± 87.92	18.055	0.164
TBil (mmol/L)	28.14 ± 10.29	24.93 ± 11.06	4.282	0.093
ChE(kU/L)	5.52 ± 2.11	5.89 ± 2.18	2.106	0.116
INR	0.98 ± 0.12	0.99 ± 0.25	1.095	0.082
First day after surgery				
PLT(× 10^9^/L)	177.37 ± 85.22	171.41 ± 90.06	26.289	0.095
ALT(U/L)	621.05 ± 481.25	529.48 ± 395.92	23.156	0.061
AST(U/L)	588.91 ± 355.21	501.89 ± 342.07	17.522	0.109
AKP(U/L)	90.14 ± 32.39	88.21 ± 36.65	11.045	0.123
GGT(U/L)	148.06 ± 52.75	92.36 ± 56.29	14.212	0.025
TBil (mmol/L)	39.12 ± 19.49	38.07 ± 18.72	8.064	0.078
ChE(kU/L)	4.03 ± 2.01	4.11 ± 2.15	2.756	0.296
INR	1.14 ± 0.86	1.21 ± 0.73	1.005	0.133

*PLT* platelet count, *ALT* alanine aminotransferase, *AST* aspartate aminotransferase, *AKP* alkaline phosphatase, *GGT* gamma glutamyl transpeptidase, *TBil* total bilirubin, *ChE* cholinesterase, *INR* international normalized ratio

### Preoperative bile culture results

As shown in Table 3, there was significant difference in the *Klebsiella pneumoniae* between biliary fistula and no biliary fistula patients (*p* = 0.015). There were no significant differences in the other pathogens of preoperative bile culture between biliary fistula and no biliary fistula patients (all *p* > 0.05).

**Table 3:** Comparison of preoperative bile culture results

Pathogens	Biliary fistula group (*n* = 33)	No biliary fistula group (*n* = 127)	*χ*^2^	*p*
Gram-positive bacteria				
*Staphylococcus aureus*	9 (27.27%)	33 (25.98%)	1.045	0.102
Hemolytic Streptococcus	5 (15.15%)	18 (14.17%)	1.191	0.092
*Enterococcus faecalis*	3 (9.09%)	15 (11.81%)	1.208	0.113
Gram-negative bacteria				
*Klebsiella pneumoniae*	12 (36.36%)	19 (14.96%)	1.266	0.015
*Pseudomonas aeruginosa*	5 (15.15%)	17 (13.39%)	1.297	0.096
*Acinetobacter baumannii*	2 (6.06%)	8 (6.29%)	1.144	0.071
*Escherichia coli*	6 (18.18%)	22 (17.32%)	1.056	0.104

### Logistic regression analysis

**Fig. 1:**
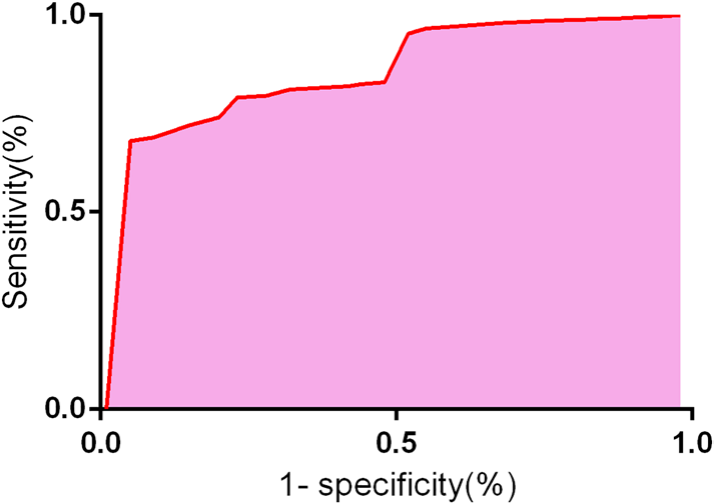
The ROC curve predicting the postoperative biliary fistulas in patients undergoing radical resection of hilar cholangiocarcinoma

Variables with clinical significance in the univariate analysis were further included in the multivariate analysis. The variable assignments of multivariate logistic regression are shown in Table 4. As indicated in Table 5, logistic regression analysis showed that age ≥ 65 years (OR 2.035, 95% CI 1.131–3.007), preoperative cholangitis(OR 1.584, 95% CI 1.081–2.361), number of biliary anastomosis ≥ 2 (OR 2.866, 95% CI 1.942–3.624), GGT on the first day after surgery ≥ 120 U/L (OR 1.823, 95% CI 1.274–2.906), preoperative bile culture for *Klebsiella pneumoniae* (OR 3.181, 95% CI 2.426–3.992) were the risk factors of postoperative biliary fistulas in patients undergoing radical resection of hilar cholangiocarcinoma (all *p* < 0.05). The ROC curve is shown in Fig. 1. The ROC curve AUC = 0.836 in this prediction model (*P* = 0.008).

**Table 4:** The variable assignment of multivariate logistic regression

Factors	Variables	Assignment
Biliary fistula	Y	Yes = 1, no = 2
Age (y)	X_1_	≥ 65 = 1, < 65 = 2
Preoperative cholangitis	X_2_	Yes = 1, no = 2
Number of biliary anastomosis	X_3_	≥ 2 = 1, < 21 = 2
GGT (U/L) on the first day after surgery	X_4_	≥ 120 = 1, < 120 = 2
Preoperative bile culture for Klebsiella pneumoniae	X_5_	Yes = 1, no = 2

*GGT* gamma glutamyl transpeptidase

**Table 5:** The logistic regression analysis on the risk factors of postoperative biliary fistulas in patients undergoing radical resection of hilar cholangiocarcinoma

Variables	β	Sx̄	OR	95% CI	*p*
Age ≥ 65y	0.227	0.103	2.036	1.131–3.007	0.043
Preoperative cholangitis	0.198	0.116	1.584	1.081–2.361	0.021
Number of biliary anastomosis ≥ 2	0.284	0.105	2.866	1.942–3.624	0.011
GGT on the first day after surgery ≥ 120 U/L	0.362	0.114	1.823	1.274–2.906	0.047
Preoperative bile culture for *Klebsiella pneumonia*e	0.327	0.191	3.181	2.426–3.992	0.013

*GGT* gamma glutamyl transpeptidase

## Discussion

Hilar cholangiocarcinoma is the main type of extrahepatic cholangiocarcinoma, accounting for 58% to 75% [19, 20]. Due to the special location of the disease and the characteristics of early invasion of blood vessels, nerves, lymphoid tissues and adjacent liver tissues, the operation for hilar cholangiocarcinoma is more difficult and complex [21, 22]. Patients with hilar cholangiocarcinoma usually require liver resection and extrahepatic bile duct resection to achieve radical cure. Previous studies [11, 23, 24] have found that the incidence of biliary fistula after such surgery is high, and biliary fistula has adverse effects on the short-term and long-term prognosis of patients. In this study, the incidence of biliary fistula after radical resection of hilar cholangiocarcinoma was 20.63%, which was lower than that reported in the previous literatures [12, 25, 26]. The reason may be that they have reported biliary fistulas with grades A, B, and C at the same time, and we only reported grade B, C cases of biliary fistula. Besides, we have found that age ≥ 65 years, preoperative cholangitis, number of biliary anastomosis ≥ 2, GGT on the first day after surgery ≥ 120 U/L, preoperative bile culture for *Klebsiella pneumoniae* are the risk factors of postoperative biliary fistulas in patients undergoing radical resection of hilar cholangiocarcinoma. Therefore, early identification, early diagnosis and early interventions of biliary fistula in high-risk patients have positive clinical significance.

Age is an important factor affecting the occurrence of biliary fistula after hilar cholangiocarcinoma. As the patient's age increases, the risk of biliary fistula after surgery is greatly increased, which may be related to the physiological characteristics of elderly patients and accompanying diseases [27]. The elderly have their special physiological and clinical characteristics. The solid organs of the elderly have different degrees of degenerative changes, the reserve function of each organ gradually declines, and the compensatory ability of some important organs also gradually declines; the immune function of the elderly declines, the ability to adapt to the internal and external environment, and the ability to resist diseases and tolerate surgery is significantly reduced [28–30]. Research statistics [31, 32] show that each elderly person suffers from changes in more than 1.6 organs on average, such as cardiovascular disease accounting for 50–60%. Surgery can exacerbate these preoperative comorbidities, and the presence of these preoperative comorbidities often complicates surgical treatment and increases the risk of serious postoperative complications [33]. Therefore, it is very important to pay attention to the influence of age on serious postoperative complications of hilar cholangiocarcinoma, and adequate preoperative evaluation and preoperative preparation should be done when formulating surgical treatment plans for elderly patients with hilar cholangiocarcinoma.

In this study, preoperative cholangitis and positive *Klebsiella pneumoniae* in preoperative bile culture may be associated with the occurrence of postoperative clinically relevant biliary fistulas. Inflammation caused by biliary tract infection can lead to bile duct wall edema and the formation of small abscesses, hinder the healing of postoperative bile duct stumps and lead to the occurrence of hepatic section or anastomotic biliary leakage [25, 34]. Previous studies [35, 36] have shown that the clinical isolation rate and drug resistance rate of *Klebsiella pneumonia*e are increasing year by year, and it has become one of the main pathogens of biliary tract infection. The choice of preventive antibiotics in our center is mainly cephalosporins, and ESBL can make bacteria resistant to most β-lactam antibiotics including cephalosporins. Bacterial virulence factors may also contribute to the development of biliary fistulas. Preoperative biliary drainage is not only the need to monitor the etiology to guide the choice of antibiotics, but also an irreplaceable means of treating acute cholangitis.

The number of biliary–enteric anastomosis was associated with the risk of postoperative biliary leakage through the biliary–enteric anastomosis. Hemihepatic resection and tri-regional resection are the most commonly used surgical methods for hilar cholangiocarcinoma. The advantages are that it is easy to obtain R0 resection, there is no large hepatic pedicle branch in the liver section, the number of bile duct openings is relatively small, and the cholangiojejunostomy is relatively simple. The disadvantage is that they are all large-scale liver resections and require high liver reserve function, so preoperative preparations such as biliary drainage are often required before surgery [37–39]. Simple perihepatectomy and other surgical procedures often have more than 3 bile duct openings, and biliary–enteric anastomosis is more difficult [40, 41]. The advantage is that they are limited liver resections, have low requirements for liver reserve function, and do not require long preoperative preparations [42]. Several studies [43–45] have demonstrated the safety and efficacy of extensive liver resection. Therefore, under the premise of the condition, a reasonable selection of large-scale liver resection can effectively reduce the number of anastomosis and the difficulty of biliary–enteric anastomosis, thereby reducing the risk of postoperative biliary fistula in patients with hilar cholangiocarcinoma.

GGT is a plasma membrane-bound glycoprotein widely distributed in mammalian tissues, and is a key enzyme in the γ-glutamyl cycle [46]. And in body fluids, the enzyme activity in serum mainly comes from the hepatobiliary system [47]. Clinical data [48] show that GGT will increase in patients with hepatocellular carcinoma, and it is positively correlated with the size and extent of cancer cells. After tumor resection, GGT can be reduced to normal, and it will increase again when it recurs. Dynamic observation of GGT may predict the curative effect and prognosis of hepatocellular carcinoma [49]. This study has found that the occurrence of postoperative clinically relevant biliary fistula is positively correlated with GGT on postoperative day 1. Previous study [50] has reported the clinical value of GGT in predicting postoperative biliary fistula in patients with hepatic hydatid disease. However, the specific mechanism of the relationship between GGT and postoperative biliary fistula has not yet been fully elucidated, which needs further investigations.

There are many limitations in this study worth considering. Firstly, as a single-center prospective study with a small sample, this study has a small sample size and may have certain selection and regional biases. Secondly, the surgical methods and perioperative management of hilar cholangiocarcinoma are still controversial in many aspects, and the differences in the diagnosis and treatment strategies of different centers will limit the development of multi-center retrospective studies. Besides, we have no further more data about the patients' comorbidities, which may influence anastomotic leakage. Therefore, it is necessary to draw more reliable conclusions by designing prospective studies in the future. Thirdly, this study failed to identify the source of the biliary fistula. Although the author's center routinely places peritoneal drainage tubes on the liver section and around the anastomosis, it is difficult to accurately determine the source of biliary fistula by detecting ascites because the drainage areas are not independent of each other. Fourthly, we did not perform microbiological analysis of the bile of all patients and preoperative drug resistances of the *Klebsiella*; those results can be useful for clinical drug use and management. Finally, no further stratified analysis of grade B and C biliary fistulas was performed in this study. Since there is no unified standard for the indications and timing of secondary surgery for postoperative biliary fistula, differences between different centers will undoubtedly affect the comparability of biliary fistula grading.

## Conclusions

In summary, this present study has found that the incidence of biliary fistula after radical resection of hilar cholangiocarcinoma is 20.63%. For patients with age ≥ 65 years, preoperative cholangitis, number of biliary anastomosis ≥ 2, GGT on the first day after surgery ≥ 120 U/L, preoperative bile culture for *Klebsiella pneumoniae*, there may have higher risk of postoperative biliary fistulas. Clinical medical workers should take early preventions and treatment strategies for these high-risk factors to reduce the occurrence of postoperative biliary fistula and improve the prognosis of patients.

## Data Availability

All data generated or analyzed during this study are included in this published article.

## Abbreviations

ISGLS: International Study Group of Liver Surgery
WBC: White blood cell
CRP: C-reactive protein
BMI: Body mass index
ASA: American Society of Anesthesiologists
PLT: Platelet count
ALT: Alanine aminotransferase
AST: Aspartate aminotransferase
AKP: Alkaline phosphatase
GGT: Gamma glutamyl transpeptidase
TBil: Total bilirubin
ChE: Cholinesterase
INR: International normalized ratio
ROC: Receiver operating characteristic
AUC: Area under the curve
